# The Wear Resistance of Reinforced Coatings Fabricated by Three Different Processes on High-Density Tungsten Alloy

**DOI:** 10.3390/ma19081605

**Published:** 2026-04-16

**Authors:** Lairong Xiao, Hongyang Chen, Fengju Zhang, Yuxiang Jiang, Siyuan Tang, Sainan Liu, Zhenyang Cai, Xiaojun Zhao

**Affiliations:** 1 School of Materials Science and Engineering, Central South University, Changsha 410083, China; xiaolr@csu.edu.cn (L.X.); hychen9171@163.com (H.C.); zhangfengju@csu.edu.cn (F.Z.); 19898826998@163.com (Y.J.); 243111019@csu.edu.cn (S.T.); 2 School of Minerals Processing and Bioengineering, Central South University, Changsha 410083, China; lsn@csu.edu.cn; 3 State Key Laboratory of Powder Metallurgy, Central South University, Changsha 410083, China

**Keywords:** tungsten alloy, laser cladding, plasma spraying, vacuum surface carburization, wear resistance

## Abstract

To address the surface wear issues of tungsten alloys in die-casting mold applications—where low hardness coupled with severe service conditions involving high-pressure impact from molten metal, thermal cycling, and component counter-friction—this study employed three techniques: laser cladding, plasma spraying, and vacuum surface carburization. Three distinct strengthening coatings were prepared on a tungsten heavy alloy (WHA) substrate. X-ray diffraction (XRD), scanning electron microscopy (SEM), a Vickers hardness tester, and block-on-ring friction and wear tests were employed to characterize the phase composition, microstructure, hardness, and wear resistance of the coatings. The results indicate that all three coatings significantly enhanced the hardness of the substrate, albeit through different strengthening mechanisms. The hardness increase in the laser-clad coating is attributed to the combined strengthening effect of rapid solidification-induced fine grains and dispersed WC particles. The enhanced hardness of the plasma-sprayed coating is due to the intrinsic hardness of WC and its dense layered structure. The carburized layer exhibits the highest hardness, resulting from the continuous WC phase formed via in situ reaction and an interface-free gradient transition with the substrate, which eliminates interfacial weak zones. Under loads of 50 N and 100 N, the plasma-sprayed coating demonstrated the best wear resistance, with wear volumes of 0.16% and 0.18% of that of the substrate, and wear depths of 4.57% and 3.50% of that of the substrate, respectively. It also exhibited the optimal load adaptability, making it a preferred solution for surface strengthening of tungsten alloy die-casting molds.

## 1. Introduction

High-density tungsten alloy (WHA) is a composite material consisting of a tungsten (W) matrix sintered with a small amount of binder elements such as Ni, Fe, Co, and Cr (with a mass percentage of less than 10%) [1]. Due to its comprehensive advantages, including high density, high melting point, low coefficient of thermal expansion, good thermal conductivity, and excellent corrosion resistance, it has been widely used in fields such as aerospace, energy, and mechanical manufacturing [2]. In die-casting mold applications, tungsten alloys are subjected to extremely demanding service conditions, where the mold must withstand the high-pressure impact of molten metal, alternating extreme thermal cycles during operation, and repeated counter-friction between mold components [3,4]. These conditions impose stringent requirements on the surface hardness and wear resistance of the mold material. However, tungsten alloys exhibit relatively low hardness (325–625 HV) [5], making it difficult to meet the long-term service demands under such harsh conditions. Therefore, effectively improving the surface hardness and wear resistance of tungsten alloys has become a critical technical challenge for expanding their applications in high-end manufacturing fields such as die-casting molds.

To address the aforementioned issue, the surface hardness and wear resistance of tungsten alloys can be enhanced by applying a strengthening coating [6,7,8]. Current methods for surface strengthening of tungsten alloys mainly include cold spraying, plasma spraying, electroplating, surface carburization, and laser cladding [9]. Among these, electroplating produces coatings that are thin and exhibit non-metallic bonding with the substrate, making them prone to cracking and spalling under prolonged service. Cold-sprayed coatings tend to have high dislocation density and residual stress, often requiring appropriate heat treatment to mitigate internal defects. Laser cladding technology rapidly melts and solidifies the coating powder on the substrate surface, producing a coating with fine grains and high toughness [10]. This technique offers advantages such as high metallurgical bonding strength, good controllability, dense coating structure, and minimal thermal deformation [11]. Jiang et al. [12] fabricated a ceramic-reinforced CoCrNiMo alloy coating using laser cladding and reported excellent metallurgical bonding between the coating and substrate, along with superior wear and corrosion resistance. Plasma spraying utilizes a high-temperature plasma arc to heat the coating powder to a molten or semi-molten state, which is then accelerated and deposited onto the substrate surface. This method is characterized by low substrate temperature, wide coating thickness range, and minimal thermal deformation; the coating also exhibits relatively low bonding strength with the substrate [13]. Huang et al. [14] deposited a tungsten coating onto a copper substrate via supersonic atmospheric plasma spraying (SAPS). The obtained coating demonstrated favorable properties, including a porosity of merely 2.3%, as well as satisfactory oxygen content, bonding performance, and hardness. The vacuum surface carburization process involves exposing the substrate to a carbon-rich atmosphere under heating, which promotes the diffusion of carbon atoms into the surface region and produces a carbide-reinforced layer possessing high hardness. This approach offers significant improvements in surface hardness while maintaining substrate toughness, producing an interface-free strengthening layer with strong adhesion [15]. Sug-Woo Jung et al. from the Korea Institute of Science and Technology [16] applied surface carburization to a W-Ni-Fe alloy and observed that with increasing carburizing temperature and time, the surface hardness of the tungsten alloy increased from approximately 500 HV to 855 HV.

In summary, existing studies have predominantly focused on the application of a single technique to a specific substrate, with a notable lack of systematic comparative investigations into three different strengthening mechanisms applied to the same WHA substrate and evaluated under the same assessment framework. Therefore, the focus of this study was to establish an integrated framework linking “preparation process—microstructure—interfacial bonding—hardness and wear resistance”, revealing the performance differences and underlying mechanisms of the three coatings under identical friction and wear conditions. By considering the service characteristics of die-casting molds, this study clarifies the advantages and limitations of the three coatings in terms of hardness enhancement, wear resistance, and load adaptability, thereby providing a systematic theoretical foundation and experimental support for the engineering selection of surface protection technologies for tungsten alloy die-casting molds.

## 2. Experimental

### 2.1. Raw Materials

The substrate material in this study consisted of a high-density tungsten alloy (96W-Ni-Fe-Co-Cr, mass%). The specific elemental composition can be found in Table 1. Given that tungsten carbide (WC) is a hard ceramic material characterized by high hardness (18–26.7 GPa at room temperature), a relatively high Young’s modulus (~700 GPa), and a comparatively low coefficient of thermal expansion (5.2 × 10^−6^ K^−1^) [17,18], it exhibits good mechanical strength, impact resistance, oxidation resistance, and chemical stability [19,20]. These properties make it commonly used in industrial applications such as cutting tools and wear-resistant components [21,22,23]. However, pure WC is relatively brittle and prone to fracture. To enhance the overall fracture toughness, metal binders such as Ni, Co, or Cr are typically added to WC powder, and the mixture is sintered to form a metal–ceramic composite [24,25].

Based on this, the feedstock materials were selected as follows: spherical Ni60-60WC powder (particle size: 200–300 mesh, 48–74 μm, Shanghai Naiou Nano Technology Co., Ltd., Shanghai, China) for laser cladding, WC-10Co-4Cr (Standard for designation: ISO 14920 [26], 15–38 μm, Zigong Cemented Carbide Co., Ltd., Zigong, China) powder for plasma spraying, and acetylene gas served as the carbon source for the vacuum surface carburization of the WHA substrate. The specific chemical composition of the powder used in the experiment is shown in Table 2, Table 3 and Table 4.

### 2.2. Coating Fabrication

Wire electrical discharge machining was employed to cut the WHA substrate into discs with dimensions of Φ26 mm × 6 mm. To remove the surface oxide layer, the substrate surfaces were sequentially ground with #240, #400, #600, #800, #1200, #1500, and #2000 abrasive papers, followed by ultrasonic cleaning in anhydrous ethanol for 5 min.

**Figure 1:**
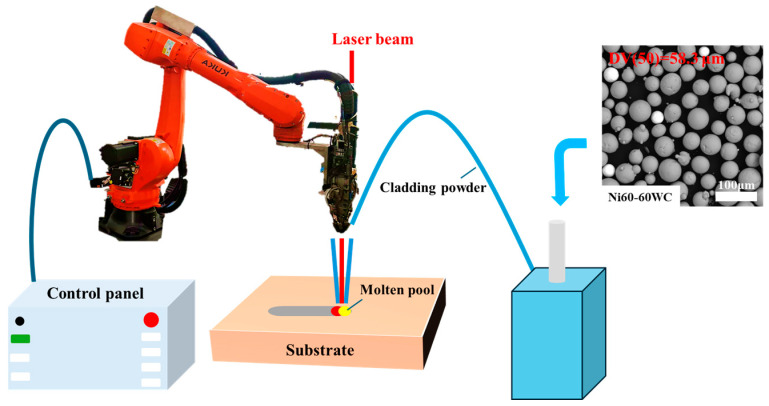
Schematic representation of the laser cladding process.

**Figure 2:**
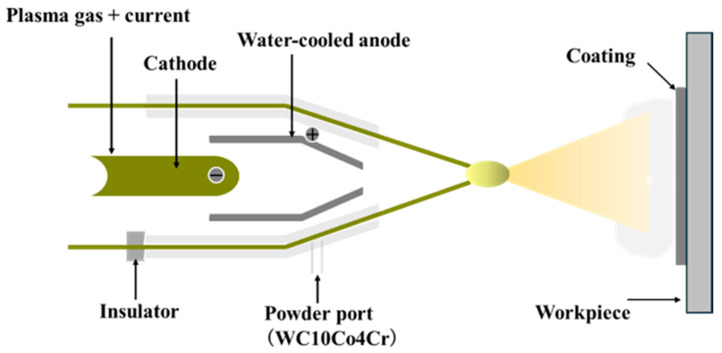
Schematic representation of the plasma spraying process.

**Figure 3:**
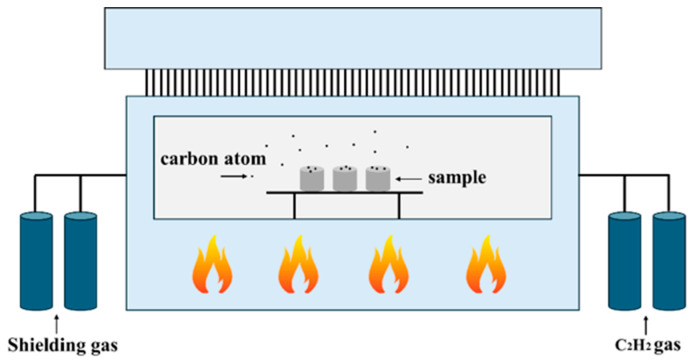
Schematic representation of the vacuum surface carburization process.

As shown in Figure 1, the laser cladding process for fabricating the coating on the WHA surface was carried out using a fiber laser device (model: YLS-6000, manufactured by IPG Photonics Corporation., Marlborough, MA, USA) equipped with a six-axis robotic arm, employing coaxial powder feeding. The process parameters were as follows: laser spot diameter of 3 mm, laser power of 1800 W, laser scanning speed of 8 mm/s, powder feeding rate of 1 r/min, an overlap ratio of 50–60%, and the laser wavelength of 1048 nm. The plasma spraying process was conducted using a HEPJet hypersonic plasma spraying system (manufactured by Beijing Jinye Longcheng Technology Co., Ltd., Beijing, China) as shown in Figure 2. The spraying parameters included a spray distance of 15 cm, a powder feed rate of 20 g/min, an argon gas flow rate of 7.0 m^3^/h, an arc current of 500 A, and an arc voltage of 120 V. Figure 3 illustrates the vacuum surface carburization process performed in a WITHJ-200 model double-chamber vacuum carburizing furnace (manufactured by China National Machinery Institute Group Beijing Electromechanical Research Institute Co., Ltd., Beijing China). The carburizing temperature was set to 1100 °C, with nitrogen and acetylene flow rates both at 12 L/min. The ratio between carburizing time and diffusion time was established as 1:2, with a total process duration of 1.5 h. Finally, the coatings prepared on the WHA surface were air-cooled to room temperature.

### 2.3. Microstructural Characterization

X-ray diffraction (XRD; Rigaku D/Max 2500, Tokyo, Japan) was used to analyze the phase composition of the coating. To reveal the microstructure, the specimens were first ground and polished with abrasive papers, and then etched for 60 s in a mixed solution of hydrogen peroxide (30% concentration) and ammonium hydroxide (28% concentration) at a volume ratio of 3:1. The microstructural morphology of the coating cross-sections was observed using a scanning electron microscope (SEM; FESEM, MIRA3 LMH, TESCAN Co., Brno, Czech Republic) fitted with an energy-dispersive X-ray spectroscopy (EDS Ultim Max 40, TESCAN Co., Brno, Czech Republic) system. Elemental analysis was performed on regions of interest. The wear scar morphology of the tested samples was characterized using a three-dimensional optical profiler (ContourX-500, Bruker Co., Billerica, MA, USA), from which three-dimensional optical profiles and cross-sectional curve data of the wear tracks were acquired.

### 2.4. Coating Performance Analysis

#### 2.4.1. Vickers Hardness

The Vickers hardness of the three coating groups and the substrate was determined on the polished surface using a computerized hardness tester (Model 200HBVS-30, Changsha Huayin Testing Instruments Co., Changsha, China) at a load of 0.5 kgf for a dwell time of 15 s in accordance with ASTM C1327 [27]. Measurements were conducted with an indentation spacing of 50 μm. On each sample, eleven indentations were applied, and the averaged values were recorded. To ensure result dependability, each group was tested three times.

#### 2.4.2. Friction and Wear Performance

Friction and wear tests were conducted on the WHA substrate and the three coating specimens prepared by different processes. First, the samples were cut into small blocks measuring 19 mm × 6 mm × 6 mm using wire electrical discharge machining. The surfaces were then ground sequentially with 80, 150, 240, 400, 600, 800, and 1000 mesh diamond grinding discs to achieve a smooth and flat finish. The friction and wear tests for the aforementioned four sample groups were performed using an HT-100 ring-on-block friction and wear testing machine (manufactured by Lanzhou Zhongke Kaihua Technology Development Co., Ltd. Lanzhou, China) under ambient temperature conditions. Applied loads were 50 N and 100 N, respectively. A Si_3_N_4_ ring with an outer diameter of 40 mm, an inner diameter of 16 mm, and a thickness of 10 mm was employed as the counterpart, and the testing machine was run at 300 r/min for 30 min. Each test was repeated three times for every sample group to minimize experimental error. During the tests, the friction coefficient for each sample group was recorded automatically by the equipment.

## 3. Results and Discussion

### 3.1. X-Ray Diffraction Analysis

**Figure 4:**
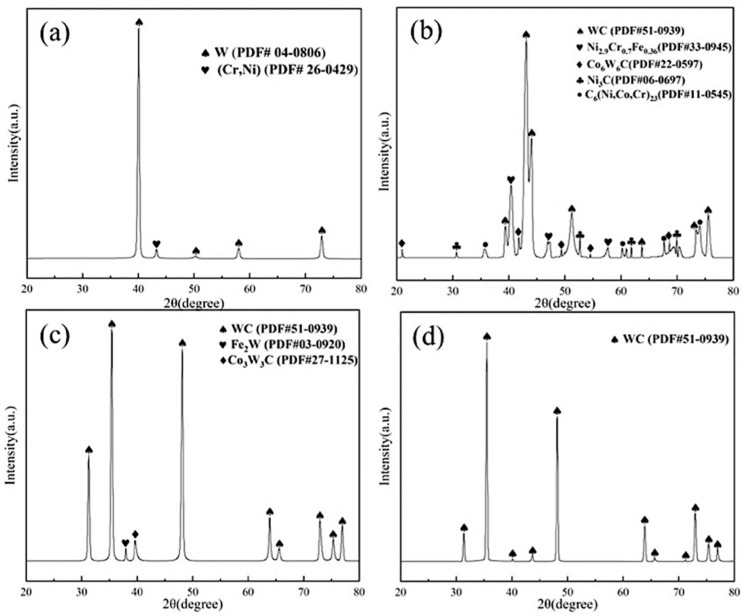
XRD patterns of the substrate and the coatings: (**a**) WHA, (**b**) laser cladding coating, (**c**) plasma spraying coating, (**d**) surface carburization coating.

Figure 4 presents the XRD patterns of the WHA substrate and the three coatings. The results indicate that, as shown in Figure 4a, the substrate material exhibits diffraction peaks corresponding to the (Cr, Ni) binder phase and the W phase. In all three coatings prepared by different methods, the WC phase was detected, which provides a fundamental guarantee for the enhanced surface hardness and wear resistance of the coatings.

Specifically, Figure 4b illustrates that the XRD results of the Ni60-60WC coating prepared by laser cladding reveal, in addition to diffraction peaks from the (Ni, Cr, Fe) intermediate phase, the presence of peaks associated with metastable phases such as C_6_(Ni, Co, Cr)_23_ and Co_6_W_6_C. This phenomenon can be attributed to the partial dissolution of WC particles during the high-temperature process. The subsequently decomposed W and C elements then interact with metallic elements from the matrix or binder, leading to the formation of the C_6_(Ni, Co, Cr)_23_ and Co_6_W_6_C metastable phases [28].

As shown in Figure 4c, the XRD results of the WC-10Co-4Cr coating prepared by plasma spraying showed additional diffraction peaks for phases such as Fe_2_W and Co_3_W_3_C. A similar mechanism is the partial decomposition of WC particles under high temperatures, where the decomposed W reacts with metallic elements (e.g., Fe from the substrate or powder) to form Fe_2_W, while WC reacts with Co to generate Co_3_W_3_C [29].

Figure 4d illustrates that the XRD results for the coating prepared by vacuum surface carburization only identified the WC phase. This is because carbon atoms generated from the decomposition of acetylene gas in the high-temperature vacuum environment preferentially reacted with W from the substrate to form WC. However, another study suggests that vacuum carburization might also produce the W_2_C phase [30]. The absence of its diffraction peaks in this analysis could be due to the limited penetration depth of XRD, which typically only probes several tens of micrometers from the material surface.

### 3.2. Microstructure

**Figure 5:**
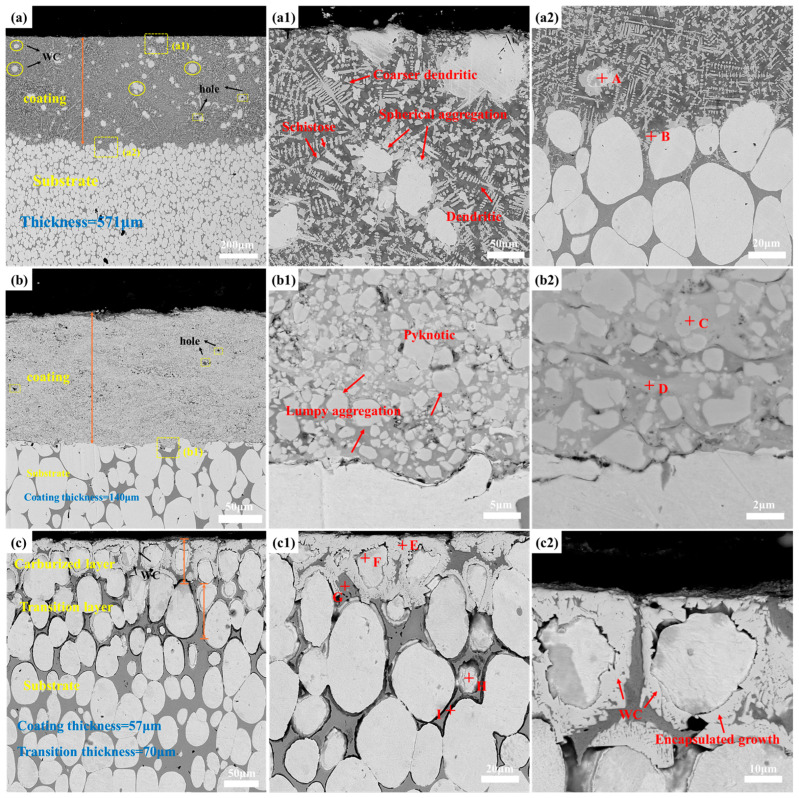
SEM images of the cross-sectional microstructure of the coatings: (**a**–**a2**) laser cladding, (**b**–**b2**) plasma spraying, (**c**–**c2**) surface carburization. Points A–I marked in (**a2**,**b2**,**c2**) represent the EDS point scan positions, and their corresponding chemical composition results are summarized in Table 5.

Figure 5 illustrates the cross-sectional microstructure of the three different coatings, and Table 5 presents the corresponding EDS point analysis results. Figure 5a presents the cross-sectional morphology of the Ni60-60WC coating fabricated by laser cladding. The overall thickness of the coating was approximately 571 μm, which is significantly greater than those of the coatings produced by the other two processes. This is attributed to the high energy of the laser beam, which simultaneously melts the metallic substrate and the cladding powder, forming a stable molten pool. The rapid solidification of the molten pool on the substrate surface results in a thick metallurgical bonding layer. Only a few small pores are present within the coating, with no through-thickness cracks, indicating a high degree of densification. This is due to the self-fluxing property of the nickel-based alloy. Under the effect of the laser, the B and Si elements in the Ni-based alloy promote deoxidation, slag formation, and wetting, significantly reducing the surface tension of the molten pool, improving its fluidity, and minimizing pore defects caused by gas entrapment during solidification [31].

**Figure 6:**
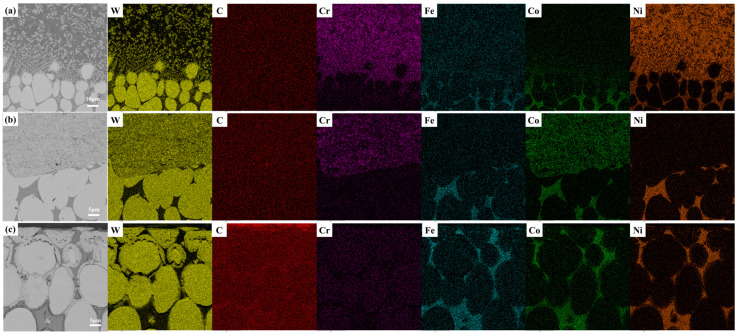
EDS elemental mapping of the three coatings: (**a**) laser cladding, (**b**) plasma spraying, (**c**) surface carburization.

Figure 5(a1) shows that the coating exhibits a typical rapidly melted and solidified microstructure, characterized predominantly by coarse dendritic crystals and eutectic structures, along with spheroidal WC reinforcing phases dispersed throughout the coating. This microstructure is a direct result of the “rapid heating–rapid solidification” process inherent to laser cladding [32]. In combination with the EDS elemental mapping results shown in Figure 6a, it can be observed that the W element was unevenly distributed within the coating, with significant enrichment in the spheroidal agglomerated phases and interdendritic regions. This is attributed to the partial dissolution and reprecipitation behavior of WC particles in the molten pool. The high energy density laser causes some WC particles to decompose into W and C elements, which subsequently reprecipitate during solidification to form WC or metastable W_2_C phases, corresponding to the spheroidal agglomerates at point A in Figure 5(a2). In contrast, Ni and Cr elements were uniformly distributed throughout the coating, forming a continuous metallic matrix phase that constitutes the tough framework of the coating. This provides the coating with a favorable basis for wear resistance, characterized by a “hard phase reinforcement + tough matrix support” mechanism.

Figure 5b shows the cross-sectional morphology of the WC-10Co-4Cr coating prepared by plasma spraying, with a coating thickness of approximately 140 μm, which is considerably lower than that of the laser cladding coating. This is because deposition during plasma spraying is achieved through layer-by-layer accumulation via mechanical interlocking and plastic deformation, and the deposition efficiency per pass is significantly lower than that of the melting and solidification accumulation in laser cladding. Consequently, the coating thickness is influenced by both the deposition amount per pass and the number of spraying passes. The coating exhibits extremely high densification, with only a few micropores and no obvious cracks. This is attributed to the high particle velocity characteristic of plasma spraying, where high-velocity particles impact the substrate and achieve tight interlocking between powder particles, effectively eliminating interlayer porosity and forming a dense coating with low porosity. The interface between the coating and the substrate exhibits mechanical interlocking characteristics.

Figure 5(b1,b2) presents the magnified morphologies, revealing that the coating consists of light gray block-like hard phases and a dark gray binder phase. Combined with the EDS elemental mapping results shown in Figure 6b, it can be observed that the W element is highly enriched in the light gray block-like regions, corresponding to the incompletely decomposed WC particles in the sprayed powder. Meanwhile, Co and Cr elements are uniformly distributed in the binder phase surrounding the WC particles, with the enrichment of Co and W in certain areas, corresponding to the dark gray region at point D. This is associated with the thermal decomposition and reaction behavior of WC particles during the spraying process: the high temperature of the plasma flame causes some WC particles to decompose into W and C elements, and the decomposition products react with the Co binder phase to form the metastable Co_3_W_3_C phase, which is uniformly distributed around the WC particles, resulting in a composite structure consisting of “WC hard phase + Co-based binder phase.” In this structure, the continuous Co-based binder phase serves to buffer stress and inhibit crack propagation.

Figure 5c shows the cross-sectional morphology of the surface carburized layer, which exhibits a clear three-layer structure: the surface carburized layer (approximately 57 μm in thickness), the intermediate transition layer (approximately 70 μm in thickness), and the underlying substrate. This is a direct result of carbon atom diffusion and in situ reaction during the gas carburizing process. In the vacuum carburizing process, acetylene decomposes into active carbon atoms at high temperatures. Driven by the concentration gradient, carbon atoms permeate into the tungsten alloy through surface adsorption and bulk diffusion, undergoing an in situ reaction with the W phase in the substrate, thereby forming a gradient carbon concentration distribution from the surface to the interior.

From the cross-sectional morphology, it can be observed that the surface carburized layer consists of in situ formed WC particles and an unreacted W matrix. The WC particles are distributed along the surfaces of the original W particles, forming a “coating growth” structure (Figure 5(c2)), with no obvious pores or crack defects. This is attributed to the uniformity of the in situ reaction, resulting in no apparent bonding defects at the coating–substrate interface. Moreover, the WC phase nucleates and grows along the surfaces of the original W particles, achieving metallurgical bonding with the substrate.

A small amount of W_2_C phase is present in the intermediate transition layer. This is because the carbon concentration exhibits a gradient distribution during the carburizing process. The carbon concentration is high at the surface, where W preferentially reacts with C to form stable WC. As carbon atoms diffuse inward, the carbon concentration in the transition layer gradually decreases, and some WC further reacts with the W in the matrix under a low-carbon potential environment to form the W_2_C phase, resulting in a transition layer structure characterized by a gradient from WC to W_2_C [33].

### 3.3. Hardness

Table 6 shows the average cross-sectional hardness of the WHA substrate and the three coated specimens. The substrate exhibited a cross-sectional hardness of 481 ± 21 HV 0.5, demonstrating the favorable baseline properties of the high-density tungsten alloy. Compared with the substrate, the hardness of all three coatings was significantly increased.

The Ni60-60WC coating produced via laser cladding exhibited a hardness of 953 ± 23 HV 0.5, roughly twice that of the substrate. This outcome can be ascribed to the exceptionally rapid heating and cooling rates characteristic of the laser cladding process, which promote non-equilibrium, rapid solidification within the molten pool and consequently yield a refined dendritic microstructure. Such grain refinement substantially improves both the strength and the hardness of the material. Furthermore, the hard WC phase was evenly distributed throughout the Ni-based alloy binder matrix, serving as a reinforcing phase that additionally enhances the coating’s hardness.

The hardness of the plasma-sprayed WC-10Co-4Cr coating was 1130 ± 58 HV 0.5, which was approximately 2.3 times that of the substrate. This coating exhibited higher hardness than the laser-clad coating, which can be attributed to the high-velocity flame jet in plasma spraying. The molten or semi-molten powder particles impact the substrate at high speed and undergo severe deformation, forming a “flattened” layered lamellar structure with tight interparticle bonding and significantly reduced porosity. The high densification contributes to the increased hardness. Furthermore, WC serves as the primary hard phase, while the Co-Cr alloy acts as the binder phase, not only encapsulating and connecting the WC particles, but also preserving their original high hardness due to the absence of significant decomposition of the WC particles.

The hardness of the WC carburized layer prepared by vacuum gas carburizing was the highest among the three, reaching 1390 ± 27 HV 0.5, which was approximately 2.9 times that of the substrate. The underlying physical mechanism is fundamentally different from the previous two cases. This is because the coating is formed via an in situ reaction. In the high-temperature vacuum environment, active carbon atoms generated by the cracking of acetylene diffuse into the tungsten alloy substrate and chemically react with W atoms to form the WC phase. The WC formed through this in situ reaction exhibits metallurgical bonding with the substrate without a macroscopic interface. Meanwhile, the WC phase in the carburized layer exhibits a continuous and dense distribution rather than discrete particles, resulting in a structure characteristic of a monolithic hard material. Furthermore, a hardness gradient transition zone exists between the carburized layer and the substrate. This gradient structure effectively alleviates stress concentration at the interface, allowing the surface hard layer to fully bear the load during hardness testing without localized fragmentation.

The physical essence of the hardness differences among the three coatings can be attributed to the differences in their strengthening mechanisms. The laser-clad coating primarily relies on grain refinement strengthening and dispersion strengthening; however, the physical composite interface between the WC particles and the matrix, along with the greater coating thickness, dilutes the volumetric effect of the hard phase. The plasma-sprayed coating fully exploits the intrinsic high hardness of the WC hard phase, and its hardness enhancement is achieved through a densified layered structure. In contrast, the carburized coating eliminates weak interfacial zones via in situ reaction, while the gradient structure ensures the effective contribution of hardness. The above mechanism analysis indicates that the hardness of a coating depends not only on the type and content of the hard phase, but is also closely related to the microstructure, interfacial bonding state, and stress state determined by the preparation process.

### 3.4. Friction and Wear Performance

**Figure 7:**
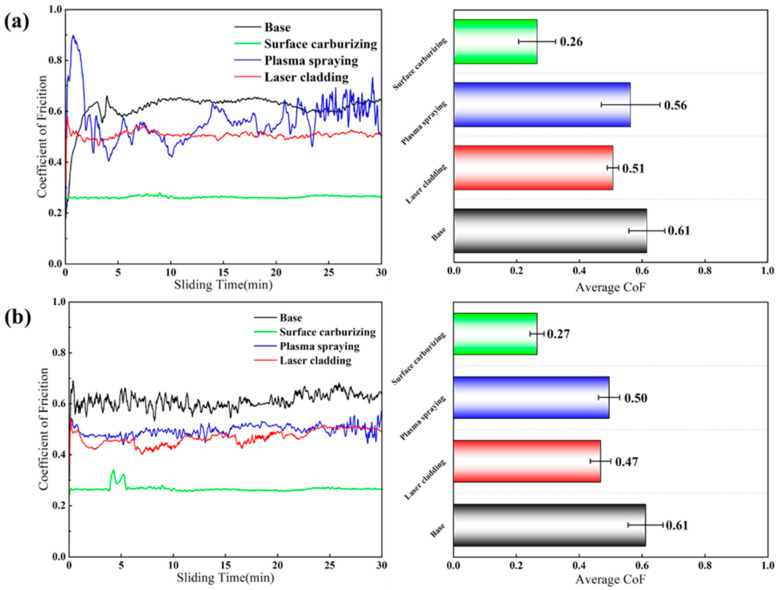
Friction coefficient (CoF) curves and average CoF values of the substrate and coatings: (**a**) under a 50 N load, (**b**) under a 100 N load.

The coefficient of friction (CoF) curves and average CoF values for the substrate and three distinct coatings, obtained under applied loads of 50 N and 100 N during friction and wear testing, are displayed in Figure 7. The findings reveal that for all specimens, the CoF rose during the early testing phase. Subsequently, as testing continued, the CoF values declined and eventually reached a stable level once the mating surfaces of the sample and counterpart became properly conformed.

Under both load conditions, the average CoF values of the substrate and the surface-carburized sample remained essentially unchanged. The average CoF values for the laser-clad and plasma-sprayed samples slightly decreased compared to those under the 50 N load but exhibited smaller fluctuations. Notably, the average CoF values for all three coating groups were significantly lower than that of the substrate, indicating that all coated samples exhibited superior wear resistance compared to the base material.

Among them, the CoF curve of the surface-carburized sample was more stable, and its average CoF value was the lowest of all four sample groups. This is attributed to the increased volume fraction of hard phases on the tungsten alloy surface due to carburization. The presence of the WC phase provided better load-bearing support, which contributed to reducing the friction coefficient and improving the uniformity of the CoF curve.

**Figure 8:**
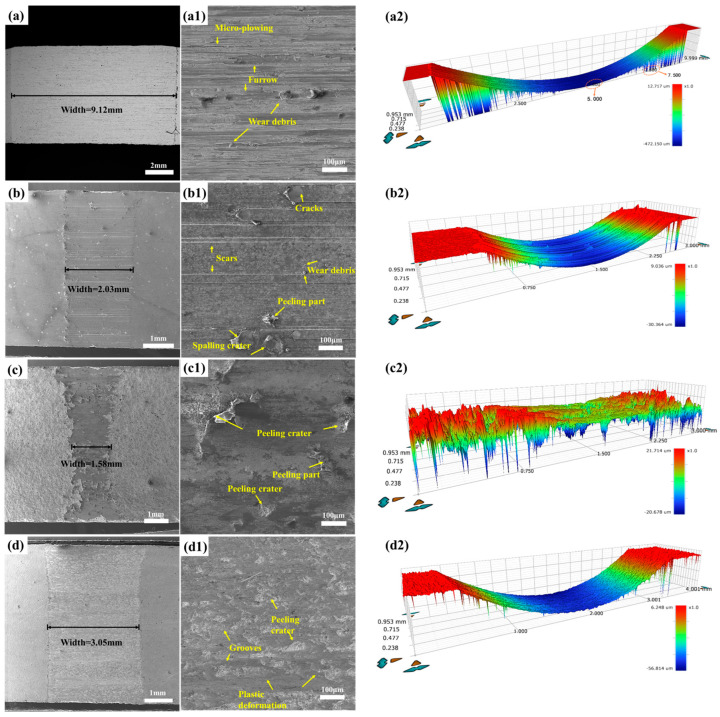
SEM images of the worn surfaces of the WHA substrate and the three coatings under a 50 N load: (**a**) WHA substrate, (**b**) laser cladding, (**c**) plasma spraying, (**d**) surface carburization; (**a1**–**d1**) are the corresponding magnified views of the areas marked in (**a**–**d**); (**a2**) 3D topography of the WHA substrate, (**b2**) laser cladding, (**c2**) plasma spraying, and (**d2**) surface carburization wear scars.

Figure 8 displays the scanning electron microscope (SEM) images alongside three-dimensional topographies depicting the worn surfaces of both the WHA substrate and the three coating specimens tested under a 50 N load. In Figure 8(a,a1), the width of the wear scar on the WHA substrate measures 9.12 mm. The worn surface exhibits pronounced evidence of severe damage, characterized by tear marks and typical long, deep, continuous grooves—features indicative of both adhesive and abrasive wear. The micro-grooves present are considerably shallower and narrower than the primary grooves, which can likely be attributed to the substantial quantity of fine wear debris produced during the friction process. Since the high-density tungsten alloy is composed of hard tungsten particles and a relatively soft binder phase (e.g., Ni, Fe), the soft binder phase tends to be preferentially worn away or detached during wear (especially abrasive wear). This loss of support leads to the dislodgement of the hard tungsten particles, thereby contributing to the relatively poor wear resistance of the tungsten alloy.

Compared to the WHA substrate, as shown in Figure 8(b,b1), the overall wear scar width of the laser-clad coating was 2.03 mm. The worn surface reveals long, penetrating cracks, along with fine scratches and scarred regions. This is attributed to the accumulation of frictional heat at the interface between the counterpart and the coating, leading to a rapid increase in temperature and mutual adhesion. Simultaneously, friction against the hard WC particles on the surface causes some particles to fracture and become exposed within the wear track. These fractured particles then act as abrasives in subsequent sliding, scratching the sample surface to form scratches and leaving behind spalling pits. The wear mechanism of the laser-clad coating is thus identified as a combination of abrasive wear and adhesive wear.

As shown in Figure 8(c,c1), the wear scar width of the plasma-sprayed coating was approximately 1.58 mm. Compared to the substrate and the laser-clad coating, its worn surface is smoother, featuring only minor spalling pits and shallower wear depths, demonstrating superior wear resistance. This enhanced performance is attributed to the high content of hard WC phase from the spray powder, which provides load-bearing support throughout the friction process. Furthermore, combined with the XRD results, the presence of the Co_3_W_3_C ceramic, known for its good self-lubricating properties, facilitates the formation of a lubricating film during friction, thereby contributing to excellent wear resistance. No significant scratches or wear debris are observed on the worn surface. The wear mechanism of the plasma-sprayed coating is identified as adhesive wear.

The wear scar width measured for the surface-carburized specimen was 3.05 mm, as illustrated in Figure 8(d,d1). A distinct oxide film is evident on the worn surface, and its layered structure displays non-conductive characteristics. Features including delamination and plastic deformation can be observed on the worn surface. According to the three-dimensional wear topography presented in Figure 8(d2), the carburized coating exhibits a slightly rough surface, which results from the oxide layer generated during the wear process. This oxide layer functions as a barrier that effectively hinders the friction counterpart from inflicting more severe wear damage on the coating, thereby enhancing its wear resistance. Consequently, the wear mechanism governing the surface-carburized coating is identified as oxidative wear.

**Figure 9:**
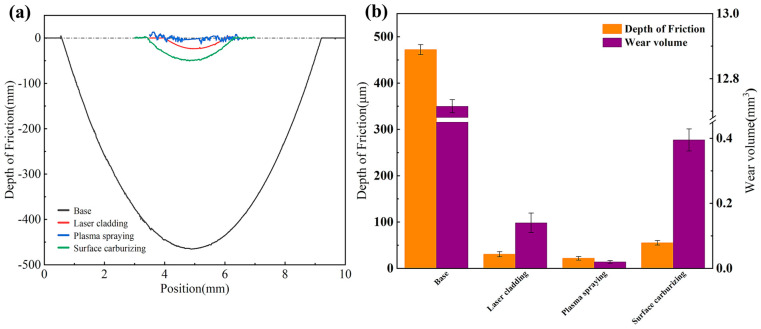
(**a**) Wear scar cross-sectional profiles of the WHA substrate and the three coating samples under a 50 N load. (**b**) Wear depth and wear volume of the WHA substrate and the three coating samples under a 50 N load.

Figure 9a shows the wear scar cross-sectional profiles of the WHA substrate and the three coating samples under a 50 N load, while Figure 9b presents their corresponding wear volumes and wear depths. The wear volume (V) during the friction and wear process can be calculated using the following formula [34]:

V = LA(1)

where A is the integral area of the wear scar cross-sectional profile, and L is the total friction and wear distance.

Relative to the substrate, all three coating specimens exhibited substantially reduced wear depth, wear width, and wear volume. The reduction observed for the laser-clad coating suggests that a suitable proportion of elongated and dendritic microstructural features contributes effectively to improved wear resistance [35]. This enhancement can be attributed to the ability of a certain number of such structures to serve as a load-bearing surface, together with the increase in coating hardness resulting from the dispersed hard WC phase, which collectively improves wear performance. However, an excessive amount of dendritic microstructure can lead to its fragmentation and spalling [36], which would increase the wear width and consequently reduce wear resistance.

The observed reduction in wear for the plasma-sprayed coating indicates that the lubricating film formed by the self-lubricating phase not only delivers effective wear resistance but also inhibits the development of grooves. Similarly, the surface-carburized coating exhibits strong wear resistance due to the shielding function of the surface oxide film. Among the three coatings, the plasma-sprayed variant demonstrated the most favorable wear behavior. Its wear depth measured 21.60 μm, equivalent to only 4.57% of that recorded for the substrate (472.16 μm), while its wear volume stood at 0.02 mm^3^, representing just 0.16% of the substrate’s value (12.72 mm^3^).

**Figure 10:**
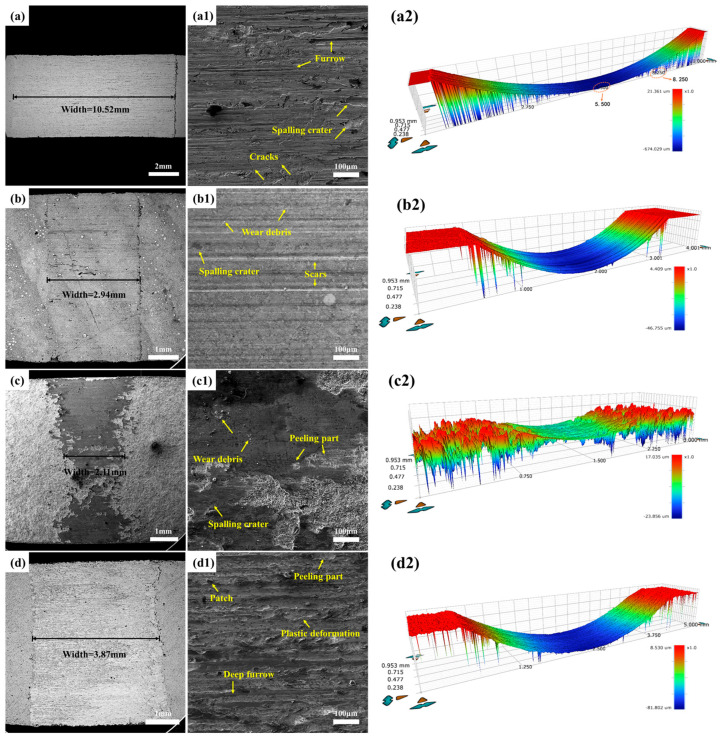
SEM images of the worn surfaces of the WHA substrate and the three coatings under a 100 N load: (**a**) WHA substrate, (**b**) laser cladding, (**c**) plasma spraying, (**d**) surface carburization; (**a1**–**d1**) are the corresponding magnified views of the areas marked in (**a**–**d**); (**a2**) 3D topography of the WHA substrate, (**b2**) laser cladding, (**c2**) plasma spraying, and (**d2**) surface carburization wear scars.

Figure 10 displays the SEM micrographs and three-dimensional topographies of the worn surfaces observed on the WHA substrate and the three coating specimens tested under a 100 N load. In Figure 10(a,a1), the grooves are deeper and more elongated, accompanied by broader tear marks. Within the torn regions, deeper furrows, enlarged spalling pits, and cracks can be identified. These findings suggest that both adhesive and abrasive wear have become considerably more severe, leading to further deterioration of the substrate.

As shown in Figure 10(b,b1), compared to the 50 N condition, the surface of the laser-clad coating exhibits more cracks under the 100 N load. This is attributed to the brittle WC ceramic phase reaching its load-bearing limit, leading to crack initiation. The generation of such defects further compromises the coating’s protective capability, resulting in decreased wear resistance.

As shown in Figure 10(c,c1), the plasma-sprayed coating still demonstrates good wear resistance under the 100 N load. Its wear width only increased by 33% compared to the 50 N condition. This is due to the presence of the self-lubricating Co_3_W_3_C ceramic phase; the solid lubricating film has not yet exceeded its shear strength limit [37] and thus remains intact under this load. A small amount of wear debris and spalling pits are visible on the worn surface. The wear mechanism for this coating remains a combination of abrasive wear and adhesive wear.

The worn surface in Figure 10(d,d1) shows deeper grooves and patches, indicating that the oxide film has essentially reached its shear stress limit under the 100 N load, leading to its failure and conversion into wear debris. Due to the coating’s extremely high surface hardness, abrasive wear is not observed on the worn surface. The wear mechanism has transitioned from oxidative wear to adhesive wear.

**Figure 11:**
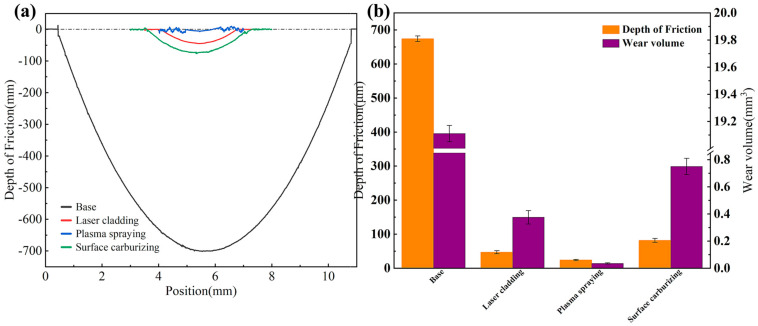
(**a**) Wear scar cross-sectional profiles of the WHA substrate and the three coating samples under a 100 N load. (**b**) Wear depth and wear volume of the WHA substrate and the three coating samples under a 100 N load.

Figure 11a shows the wear scar cross-sectional profiles of the WHA substrate and the three coating samples under a 100 N load. Figure 11b presents their corresponding wear volumes and wear depths. The wear amounts for all three coating samples remain substantially lower than those of the substrate. It is worth noting that the plasma-sprayed coating sample exhibited the smallest wear depth and also the lowest wear volume (0.035 mm^3^), which was only 0.18% of the substrate’s (19.11 mm^3^).

### 3.5. Wear Mechanisms and Transition

**Figure 12:**
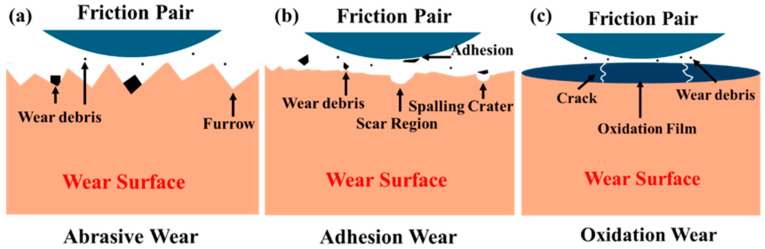
Schematic diagram of wear mechanisms: (**a**) abrasive wear, (**b**) adhesive wear, (**c**) oxidative wear.

In the process of friction and wear testing conducted on both the substrate and coating samples, various wear mechanisms were observed alongside the progression of damage. The present investigation focused on three principal wear mechanisms: abrasive, adhesive, and oxidative wear. These mechanisms are schematically illustrated in Figure 12.

Abrasive wear represents a frequently observed mechanism within material wear processes, typically characterized by a hard counterpart cutting into the specimen surface, which generates debris as friction occurs and ultimately results in the appearance of abrasive scratches [38]. Figure 12a illustrates the typical features of abrasive wear, such as grooves and adjacent plastic deformation buildup. Adhesive wear also frequently occurs as a mechanism in material wear processes. This mechanism arises when adhesion between the contacting surfaces of the material and its counterpart leads to partial detachment of material particles, followed by separation [39]. Indicative signs of adhesive wear, as depicted in Figure 12b, consist of spalling pits and regions exhibiting scratching. Oxidative wear constitutes a type of chemical wear, in which the surface constituents of a material undergo reaction with atmospheric oxygen [40]. This represents a classic instance of chemically-induced wear, frequently leading to the development of an oxide layer on the material surface, as shown in Figure 12c.

Under both 50 N and 100 N loads, the substrate and coating materials demonstrated comparable wear mechanisms as well as similar trends in wear resistance. Given the presence of ceramic constituents within the coatings, this observation can be interpreted through the lens of wear transition theory, a concept rooted in ceramic wear research [41]. According to this framework, the friction and wear behavior of ceramics is categorized into three distinct stages based on the degree of wear severity: mild wear, moderate wear, and severe wear. The transition in wear mechanisms that occurs between these stages is defined as wear transition. Key parameters influencing wear volume include applied load, sliding speed, test duration, and temperature, among others [42,43,44].

For the WHA substrate material, the primary reason for the transition from mild to severe wear is the increase in applied load. Without the protection of a coating, the high-load friction induces deeper grooves and tears in the wear zone, further leading to large spalling pits and a consequent increase in wear volume.

In contrast, all three coating materials demonstrated excellent wear resistance. However, under high load, the laser-clad coating experienced fracture of the WC ceramic phase as it exceeded its load-bearing limit, resulting in pits and surface cracks, which degraded its wear performance. For the surface-carburized coating, the increased load generated higher radial forces causing cracks in the surface oxide film. As wear time progressed, these cracks propagated deeper and outward. Fracture induced by these cracks led to coating damage under high contact stress, reducing its wear resistance. The solid lubricating film in the plasma-sprayed coating, even under the 100 N load, did not reach its shear limit, effectively protecting the substrate and exhibiting the most superior wear resistance.

## 4. Conclusions

This study employed three processes—laser cladding, plasma spraying, and vacuum surface carburization—to prepare a Ni60-60WC cladding layer, a WC-10Co-4Cr coating, and a WC-based in situ carburized layer on a WHA substrate, respectively. The microstructures and friction and wear properties of the substrate and the three coatings were systematically analyzed and compared. The main conclusions are as follows:

(1) Three strengthening coatings significantly enhanced the surface hardness of the WHA substrate. The vacuum gas carburized layer, which formed a continuous WC phase via in situ reaction and a gradient transition without a distinct interface, achieved the highest hardness of 1390 HV 0.5—approximately 2.9 times that of the substrate. In comparison, the plasma sprayed coating reached 1130 HV 0.5, and the laser clad coating 953 HV 0.5. These results indicate that an in situ metallurgical bonding interface is more effective than the mechanical/metallurgical composite interface of externally applied coatings in leveraging the strengthening phase to improve hardness.
(2) Three coatings exhibited favorable friction-reducing and wear resistant properties, though via different mechanisms. Compared with the WHA substrate, their friction coefficient, wear track dimensions, and wear volume were substantially reduced. The laser clad coating relies on its large thickness and uniformly dispersed WC hard phase, despite localized brittle spalling. The plasma sprayed coating offers stable friction reduction due to good mechanical bonding and high hardness. Although thinner, the carburized layer eliminates the macroscopic interface via an in situ metallurgical bond and forms a hardness gradient, also achieving excellent wear resistance. Thus, the three coatings effectively protected the WHA substrate through distinct mechanisms.
(3) Under both 50 N and 100 N loads, the plasma sprayed WC-10Co-4Cr coating exhibited the best wear resistance, showing the lowest wear volume, track width, and depth. It maintained excellent performance under both low and high loads, with load-induced wear mechanism transition being the main factor affecting wear loss. In contrast, the laser clad coating suffered increased brittle spalling under high load, and the carburized layer lost its advantage due to limited thickness. Therefore, the plasma sprayed coating offered the best load adaptability and overall wear resistance under the investigated conditions.

## Figures and Tables

**Table 1:** Chemical composition of the WHA substrate.

Element	Ni	Fe	Co	Cr	W
Content (mass%)	1.96	0.86	0.69	0.18	Bal.

**Table 2:** Chemical composition of the Ni60 powder.

Element	C	B	Si	Cr	Fe	Ni
Content (mass%)	0.71	3.22	4.05	16.12	3.27	Bal.

**Table 3:** Powder composition for the laser cladding.

Composition	Content (Mass%)	
	Ni60	WC
Ni60-60WC	40	60

**Table 4:** Powder composition for the plasma spraying.

Composition	Content (Mass%)		
	WC	Co	Cr
WC-10Co-4Cr	86	10	4

**Table 5:** Point composition analysis (at.%) of the coating microstructures shown in Figure 5.

Point	Element						Possible Phase
	C	Cr	Fe	Co	Ni	W	
A	53.75	-	0.17	0.30	0.95	44.83	WC
B	46.54	4.40	1.04	3.36	13.26	31.42	WC + W_2_C
C	46.03	2.32	0.47	4.80	0.60	45.78	WC
D	43.50	15.60	-	24.61	0.10	16.20	WC + Co_3_W_3_C
E	74.41	-	0.05	-	-	25.53	WC + W_2_C
F	66.29	-	0.11	0.12	0.37	33.11	W_2_C
G	41.66	-	8.40	13.99	31.44	4.51	(Fe, Co, Ni)C
H	61.13	-	0.70	0.33	0.54	37.30	W_2_C
I	88.58	-	1.60	1.98	5.34	2.50	(Fe, Co, Ni)C

**Table 6:** Cross-sectional Vickers hardness of the substrate and the three coating samples.

Samples	Cross-Sectional Vickers Hardness (HV 0.5)										Average	Standard Deviation
Base	465	513	467	474	486	511	488	466	492	448	481	21
Laser cladding	963	953	934	989	929	975	983	941	928	935	953	23
Plasma spraying	1136	1125	1208	1188	1145	1192	1148	1078	1075	1025	1130	58
Surface carburizing	1394	1408	1345	1379	1422	1438	1375	1401	1368	1370	1390	27

## Data Availability

The original contributions presented in this study are included in the article. Further inquiries can be directed to the corresponding authors.

## References

[1] Liu K., Zhang L., Cao L., Wang T., Fu J., Wang J., Zhang J., Yang X., Cao Y., Liu B. (2025). Binder jetting additive manufacturing of a 95W-3.5Ni-1.5Fe tungsten heavy alloy: Enhanced ductility and dynamic deformation mechanisms. Mater. Sci. Eng. A.

[2] Yuan Y., Yu H., Han Y., Fang W. (2025). Synergistic strengthening-toughening strategy for additively manufactured tungsten heavy alloys via alloying element-induced hierarchical microstructures. Chem. Eng. J..

[3] Levin Z.S., Ted Hartwig K. (2015). Hardness and microstructure of tungsten heavy alloy subjected to severe plastic deformation and post-processing heat treatment. Mater. Sci. Eng. A.

[4] Sattar H., Jielin S., Ran H., Imran M., Ding W., Das Gupta P., Ding H. (2020). Impact of microstructural properties on hardness of tungsten heavy alloy evaluated by stand-off LIBS after PSI plasma irradiation. J. Nucl. Mater..

[5] Zuo Z., Wang X., Wang Y., Wang B., Wei S., Dai S. (2025). Multi-objective optimization design of tungsten alloy long-rod armor-piercing projectile penetrating ceramic-rubber composite armor. Eng. Comput..

[6] Adesina A.Y., Iqbal Z., Al-Badour F.A., Gasem Z.M. (2019). Mechanical and tribological characterization of AlCrN coated spark plasma sintered W–25%Re–Hfc composite material for FSW tool application. J. Mater. Res. Technol..

[7] Jiang Y., Yang J.F., Liu R., Wang X., Fang Q. (2014). Oxidation and corrosion resistance of WC coated tungsten fabricated by SPS carburization. J. Nucl. Mater..

[8] Yoon J.K., Lee K.W., Chung S.J., Shon I.-J., Doh J.-M., Kim G.-H. (2006). Growth kinetics and oxidation behavior of WSi_2_ coating formed by chemical vapor deposition of Si on W substrate. J. Alloys Compd..

[9] Su J., Li A., Ruan Y., Qiu X., Liang J. (2024). Repair of tungsten heavy alloy die casting molds with laser cladded CoCrFeNiWx high entropy alloy Coatings: Microstructure, wear properties and tempering stability. Eng. Fail. Anal..

[10] Siddiqui A.A., Dubey A.K. (2021). Recent trends in laser cladding and surface alloying. Opt. Laser Technol..

[11] Garzón-Manjón A., Christiansen L., Kirchlechner I., Breitbach B., Liebscher C., Springer H., Dehm G. (2019). Synthesis, microstructure, and hardness of rapidly solidified Cu-Cr alloys. J. Alloys Compd..

[12] Jiang D., Cui H., Chen H., Zhao X., Ma G., Song X. (2021). Wear and corrosion properties of B_4_C-added CoCrNiMo high-entropy alloy coatings with in-situ coherent ceramic. Mater. Des..

[13] Liu K., Guo M., Ye G., Wang Y., Fan W., Du J., Cao Y., Zhang L. (2026). Advances in plasma spraying technology for coatings: From particle flight to splat formation. Appl. Mater. Today.

[14] Huang J.J., Wang F., Liu Y., Jiang S., Wang X., Qi B., Gao L. (2011). Properties of tungsten coating deposited onto copper by high-speed atmospheric plasma spraying. J. Nucl. Mater..

[15] Wang G.h., Qu S.g., He R.l., Hu K., Li X.-Q. (2016). Effect of carburization on microstructure and rolling contact fatigue property of 95W–3.4Ni–1.6Fe heavy alloy. Trans. Nonferrous Met. Soc. China.

[16] Jung S.W., Kang S.J.L., Kim D.K., Lee S., Noh J.-W. (1999). Effect of surface carburization on dynamic deformation and fracture of tungsten heavy alloys. Metall. Mater. Trans. A.

[17] Li G., Li Y., Wang Y. (2026). Microstructure and high-temperature tribological properties of WC/Fe functionally graded coating fabricated by laser directed energy deposition. Tribol. Int..

[18] Wang Y., Gu B., Liu C., Xu G., Guo H., Wang M., Sun S., Liu F., Pan L. (2025). Enhanced High-Temperature Tribological Performance of laser direct energy deposition WC/Inconel 718 Composites: Microstructure, Mechanisms, and Compositional Optimization. Tribol. Int..

[19] Yang Z., Wang R., Li H., Yang Y., Liu Y., He W. (2026). Effect of WC on grain refinement of binder alloy in EB-PBFed CuNiFeSnTi/WC/diamond composites. Int. J. Refract. Met. Hard Mater..

[20] Bao S., Cui Y., He Q., Wang A., Wang W., Fu Z. (2026). Microstructure and mechanical properties of ultrafine WC hard alloy prepared by high-energy ball milling using ZrO_2_ beads. Ceram. Int..

[21] Piechowiak-Osak D., Kotkowiak M., Makuch N., Miklaszewski A. (2026). Influence of WC content on tribology behaviour and nanomechanical properties of NiAl-WC composites. Wear.

[22] Wang S., Wang Z., Liu L., Tang D., Yin C., Shangguan X. (2026). Preparation of fine-grained WC-4Co cemented carbides cutting tools with engineered surface micro-features by SPS and performance analysis. Int. J. Refract. Met. Hard Mater..

[23] Da Silva F.A.V., Franzão H.S., Silveira T.F.S., Outeiro J. (2025). Prediction of surface modifications induced by orthogonal cutting of IN718 using uncoated WC-Co and PCBN cutting tools. Manuf. Lett..

[24] Müller D., Konyashin I., Ries B., Pötschke J., Michaelis A. (2026). Novel WC-based cemented carbides with Fe-Cr-V binders with improved fracture toughness for neutron shielding applications. Int. J. Refract. Met. Hard Mater..

[25] Su Y., Li Q., He D., Zhang J. (2026). Lightweight diamond/WC–Co composites achieve synergistic hardness-toughness enhancement via high-pressure sintering. Int. J. Refract. Met. Hard Mater..

[26] (2023). Thermal Spraying—Spraying and Fusing of Self-Fluxing Alloys.

[27] (2019). Standard Test Method for Vickers Indentation Hardness of Advanced Ceramics.

[28] Li Y., Jia Y., He C., Zhao Y., Wang Z., He Z., Zhou S., Zhao J. (2026). Investigation on microstructure and properties of WC Particle-Reinforced Iron-Based coatings fabricated by Field-Assisted laser cladding. Opt. Laser Technol..

[29] Cui S., Miao Q., Liang W., Xu Y., Li B. (2017). Comparative analysis of tribological behavior of plasma- and high-velocity oxygen fuel-sprayed WC-10Co-4Cr coatings. Ind. Lubr. Tribol..

[30] Li W., Li A., Liang Y., Zhang Z., Cui J. (2020). Effect of vacuum carburizing on surface properties and microstructure of a tungsten heavy alloy. Mater. Res. Express.

[31] Yang H.Y., Wang Z., Chen L.Y., Shu S.-L., Qiu F., Zhang L.-C. (2021). Interface formation and bonding control in high-volume-fraction (TiC+TiB_2_)/Al composites and their roles in enhancing properties. Compos. Part B Eng..

[32] Tobar M.J., Álvarez C., Amado J.M., Rodríguez G., Yáñez A. (2006). Morphology and characterization of laser clad composite NiCrBSi–WC coatings on stainless steel. Surf. Coat. Technol..

[33] Zhang Q., Yang J., Deng N., Cheng Y., Ding Y., Chen Z., Yang B., Liang S. (2023). Effect of carburized time on microstructure and properties of W Cu composites fabricated by vacuum pulse carburization. Int. J. Refract. Met. Hard Mater..

[34] Zhou K., Pei H., Xiao J., Zhang L. (2022). Micro-scratch behavior of WC particle-reinforced copper matrix composites. Rare Met..

[35] Fu L., Yang J., Bi Q., Liu W. (2011). Improved Wear Resistance of Dendrite Composite Eutectic Fe-B Alloy. J. Tribol..

[36] Jalali Ms Zarei-Hanzaki A., Mosayebi M., Abedi H., Malekan M., Kahnooji M., Farabi E., Kim S.-H. (2023). Unveiling the influence of dendrite characteristics on the slip/twinning activity and the strain hardening capacity of Mg-Sn-Li-Zn cast alloys. J. Magnes. Alloys.

[37] Zhou S., Jian R., Liang Y.J., Zhu Y., Wang B., Wang L., Wang L., Ren Y., Xue Y. (2021). High susceptibility to adiabatic shear banding and high dynamic strength in tungsten heavy alloys with a high-entropy alloy matrix. J. Alloys Compd..

[38] Zhou K., Xiao G., Xu J., Huang Y. (2023). Wear evolution of electroplated diamond abrasive belt and corresponding surface integrity of Inconel 718 during grinding. Tribol. Int..

[39] Mishina H., Hase A. (2019). Effect of the adhesion force on the equation of adhesive wear and the generation process of wear elements in adhesive wear of metals. Wear.

[40] Sarıkaya M., Gupta M.K., Tomaz I., Pimenov D.Y., Kuntoğlu M., Khanna N., Yıldırım Ç.V., Krolczyk G.M. (2021). A state-of-the-art review on tool wear and surface integrity characteristics in machining of superalloys. CIRP J. Manuf. Sci. Technol..

[41] Xiong F., Manory R.R. (1999). The effect of test parameters on alumina wear under low contact stress. Wear.

[42] Kim J.H., Choi S.G., Kim S.S. (2019). A Fracture Mechanics Approach to Wear Mechanism of Ceramics Under Non-conformal Rolling Friction. Int. J. Precis. Eng. Manuf..

[43] Lou M., Chen X., Xu K., Deng Z., Chen L., Lv J., Chang K., Wang L. (2021). Temperature-induced wear transition in ceramic-metal composites. Acta Mater..

[44] Bosman R., Schipper D.J. (2010). On the transition from mild to severe wear of lubricated, concentrated contacts: The IRG (OECD) transition diagram. Wear.

